# Parsing Heterogeneous Striatal Activity

**DOI:** 10.3389/fnana.2017.00043

**Published:** 2017-05-16

**Authors:** Kae Nakamura, Long Ding

**Affiliations:** ^1^Department of Physiology, Kansai Medical UniversityHirakata, Osaka, Japan; ^2^Department of Neuroscience, University of PennsylvaniaPhiladelphia, PA, United States

**Keywords:** basal ganglia, striatum, dopamine, saccade, primate, reward, decision making, rodents

## Abstract

The striatum is an input channel of the basal ganglia and is well known to be involved in reward-based decision making and learning. At the macroscopic level, the striatum has been postulated to contain parallel functional modules, each of which includes neurons that perform similar computations to support selection of appropriate actions for different task contexts. At the single-neuron level, however, recent studies in monkeys and rodents have revealed heterogeneity in neuronal activity even within restricted modules of the striatum. Looking for generality in the complex striatal activity patterns, here we briefly survey several types of striatal activity, focusing on their usefulness for mediating behaviors. In particular, we focus on two types of behavioral tasks: reward-based tasks that use salient sensory cues and manipulate outcomes associated with the cues; and perceptual decision tasks that manipulate the quality of noisy sensory cues and associate all correct decisions with the same outcome. Guided by previous insights on the modular organization and general selection-related functions of the basal ganglia, we relate striatal activity patterns on these tasks to two types of computations: implementation of selection and evaluation. We suggest that a parsing with the selection/evaluation categories encourages a focus on the functional commonalities revealed by studies with different animal models and behavioral tasks, instead of a focus on aspects of striatal activity that may be specific to a particular task setting. We then highlight several questions in the selection-evaluation framework for future explorations.

## Introduction

The striatum, an input channel of the basal ganglia, receives massive projections from the cortex and shows funnel-like connections to other subcortical regions. The striatum is also noted for heterogeneity in neuronal responses. To give a very narrow set of examples, in the sensory domain, striatal neurons respond to stimuli of all modalities (e.g., visual and auditory (Hikosaka et al., 1989b), somatosensory (Schneider and Lidsky, 1981), olfactory (Wang et al., 2013)); in the motor domain, they become active before, during and after skeletal and oculomotor movements (Hikosaka et al., 1989a; Alexander and Crutcher, 1990b; Romo and Schultz, 1992; Schultz and Romo, 1992); in the motivational domain, they respond to appetitive and aversive stimuli (Hikosaka et al., 1989c; Delgado et al., 2008); and in higher cognitive domains, they modulate their activity depending on task context, outcome expectation, choice and learning status (Apicella et al., 1992; Hollerman et al., 1998; Kawagoe et al., 1998; Lauwereyns et al., 2002; Takikawa et al., 2002; Brasted and Wise, 2004; Barnes et al., 2005; Pasupathy and Miller, 2005; Ding and Hikosaka, 2006; Lau and Glimcher, 2007).

Given the diverse types of responses and connectivity, two theories have been especially influential for understanding the neural computations in the striatum (and the basal ganglia in general). Alexander et al. (1986) proposed that the basal ganglia are organized as parallel, functional modules at the macroscopic level, with the modules sharing similar architecture but, when applied to different inputs, mediating different cognitive, motor and limbic functions. Built upon the idea of modular organization, Redgrave et al. (1999) proposed that the basal ganglia are well-suited to perform selection of relevant quantities to drive behaviors. Implicitly, the heterogeneity of striatal activity may simply reflect the diversity of behaviorally relevant sensory inputs, internal states and motor outputs.

Here we review some recent monkey and rodent neurophysiological data and suggest that the heterogeneity of striatal activity also reflect the presence of multiple computational components that can be used to serve a general-purpose selection machinery. We propose that, within a macroscopic module, striatal activity reflects signals related to implementation and evaluation of the selection process. More specifically, we consider signals that occur before an action and differentiate between alternative actions to be related to selection; we consider signals that reflect expected and/or received outcome without differentiating between alternative actions to be related to evaluation. To illustrate this idea, we focus on activity of putative striatal projection neurons for two types of behavioral tasks. For the first type, reward outcome is manipulated such that subjects select and/or modulate their actions based on the expected outcome of available alternatives. For the second type, properties of the visual stimulus are manipulated such that subjects select and/or modulate their actions based on available visual evidence. As we describe below, neurons in the caudate (monkeys) and dorsomedial striatum (rodents) show diverse task-related modulation, with shared features that may reflect implementation and evaluation for selection processes that are involved in both types of tasks.

## Striatal Activity Reflects Quantities Necessary for Selection

**Figure 1:**
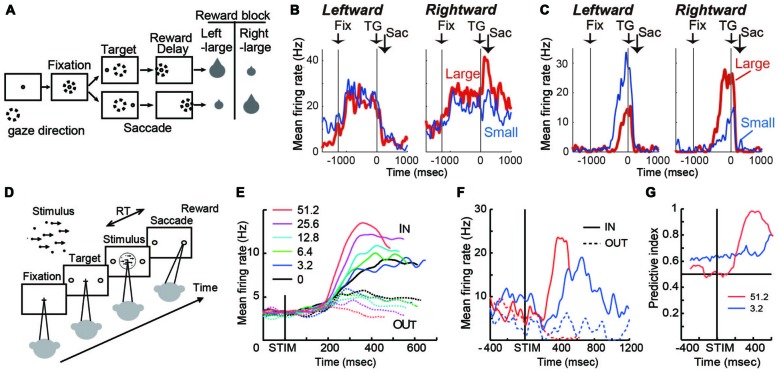
**(A)** Visually guided saccade task with an asymmetric reward schedule. After fixating on the central fixation point (FP), a target cue appeared immediately on either the left or right, to which the monkey made a saccade to receive a liquid reward. The dotted circles indicate the direction of gaze. In a block of 20–28 trials (e.g., left-large block), one target position (e.g., left) was associated with a large reward, and the other position (e.g., right) was associated with a small reward. The position-reward contingency was then reversed (e.g., right-large block). **(B)** An example dorsal caudate neuron showing a target-direction effect (right-target dominant) after target onset until reward delivery. This neuron also showed a reward-size effect (right-large-reward dominant). Spike density functions (top) and raster plots in the chronological order are aligned to target onset (left, TG on) and reward onset (right, RW on). Red: large-reward trials; blue: small-reward trials; green dots: FP onset; black dots: saccade onset; light blue dots: reward onset and offset. Dots for reward offset are only visible for large-reward trials. **(C)** An example dorsal caudate neuron showing a reward-direction effect. Note that this neuron showed stronger pre-target activity for the right-large block. **(D)** The motion discrimination task. The monkey decides the global motion direction of a random-dot kinematogram and then at a self-determined time, make a saccade to one of two choice targets. Saccades to the target in the direction of coherent motion are followed by juice reward. **(E)** Population average of evidence accumulation activity aligned on stimulus onset for correct trials (truncated at median reaction time (RT) after excluding activity 100 ms before saccade onset). Solid lines, trials to the neurons’ preferred direction (IN trials); dashed lines, trials away from preferred direction (OUT trials). Coherence levels are indicated by colors. **(F)** Activity of an example neuron before and during motion viewing. Blue, 3.2%; red, 51.2% motion coherence. Note that the activity *before* stimulus onset was different between trials with different final choices (solid vs. dashed lines) at 3.2% coherence, but it was not at 51.2% coherence. **(G)** Time course of the predictive index, which quantifies how well an ideal observer can predict the final choice based on neural activity. Before stimulus onset, it was significantly larger than chance (0.5) for low motion-strength trials (e.g., 3.2% coherence) and at chance for high motion-strength trials (e.g., 51.2% coherence); after stimulus onset, the pattern reversed, with the predictive index increasing sharply for high motion-strength trials.

The caudate nucleus has been extensively studied in relation to neural representation of action value for selection. A prominent paradigm for such neurophysiological studies is the asymmetric reward saccade task (Figure 1A). On a trial, a subject makes a saccade to a visual target to receive a reward. The saccade target is randomly placed at one of two locations. In a block of trials, one target location (e.g., left) is always associated with a large reward, while the other (e.g., right) is always associated with a small reward. The block design allows the monkey to maintain information about the current reward context information (i.e., which location is more desirable).

On this task, many caudate neurons show reward-dependent modulation of target-related activity, which may reflect their roles in implementing selection (Kawagoe et al., 1998; Lauwereyns et al., 2002; Takikawa et al., 2002; Samejima et al., 2005; Ding and Hikosaka, 2006; Kobayashi et al., 2007). For example, the neuron in Figure 1B was activated more by target presentation at the right than the left (Nakamura et al., 2012). In addition, for the same right target presentation, its response was further augmented if the right target is associated with the large reward. This target-direction dependent modulation in activity is observed in around 15% of analyzed caudate neurons (Nakamura et al., 2012). Such activity may confer useful information for selection based on the “value” of the targets.

Because reward context information is constant in a block of trials, monkeys exhibit robust behavioral bias toward the larger-reward target, as reflected in biased choice and reaction time (RT) in free-choice and forced-choice versions of the task, respectively (Coe et al., 2002; Lauwereyns et al., 2002). The RT bias can be modulated by the duration of the foreperiod before target appearance, suggesting that the selection process uses not only neural signals reflecting the value of the target, but also reward context-dependent signals present in the foreperiod in the absence of any target information (Ding and Hikosaka, 2007). Remarkably, consistent with the foreperiod duration-modulated RT bias, some caudate neurons show reward context-modulated activity during the foreperiod, with the difference between reward contexts increasing with time before target presentation (Lauwereyns et al., 2002; Takikawa et al., 2002; Ding and Hikosaka, 2006; Nakamura et al., 2012). For example, the neuron in Figure 1C gradually ramped up its activity, reaching a much higher level *before* target onset in blocks when the contralateral target is paired with the larger reward. This type of activity may contribute to reward context-based bias in the selection process to favor one action in the absence of additional input. Such block-wise, reward context-specific signal that emerges before the appearance of a visual target, is prevalent in the dorsal/central portion of caudate (Nakamura et al., 2012).

The asymmetric reward task uses salient, unambiguous visual targets and manipulates their reward associations. In a complementary paradigm, the random-dot visual motion direction discrimination task uses equal reward associations and instead manipulates the discriminability of the visual stimulus. On a trial, a subject is presented with a random-dot kinematogram and asked to make a saccade to the target congruent with the motion direction of the dots (Figure 1D). The direction and motion strength (expressed as the percentage of dots moving coherently) are randomized across trials. Behavioral performance on this task, by human and monkey subjects, can be well accounted for by a theoretical framework, in which noisy motion evidence is accumulated over time into a decision variable to guide the subject’s decision about motion direction (equivalent to the saccade target). On this task, a subset of caudate neurons show patterns of choice, motion strength and time modulation consistent with predictions of a decision variable in the accumulation framework (Figure 1E; Ding and Gold, 2010). For example, after motion stimulus onset, the average activity gradually diverged for trials ending with different choices (solid vs. dashed lines); the amount of divergence increased with time; and the amount of divergence was larger for trials with higher motion strength (e.g., red vs. blue, Figure 1E). Such activity may reflect and/or contribute to formation of the decision variable, the basis for selection.

In addition to the stimulus-dependent activity modulation, a small subset of caudate neurons showed bias-like activity before motion stimulus onset, reminiscent of the pre-target reward bias-related activity on the asymmetric reward task (Figure 1F; Ding and Gold, 2010). In the context of motion discrimination, bias-related activity is expected to influence the final choice more when only weak evidence is available and be overridden when strong evidence is available. The example neuron in Figure 1F conforms to such expectations: the activity was different *before* stimulus onset, between trials with different final choices (solid vs. dashed lines) at 3.2% coherence, but not at 51.2% coherence. The contribution of such activity to the final choice can be quantified by a predictive index, which measures how well an observer can predict the final choice based on neural activity. As in Figure 1G, the predictive index was significantly larger than chance (0.5) for low motion-strength trials and hovers around chance for high motion-strength trials, indicating significant influence of the bias-related activity on the final choice when evidence is weak. For comparison, after stimulus onset, the pattern reversed, with the predictive index increasing sharply for high motion-strength trials. These types of activity tended to be observed in dorso-lateral layers of the rostral caudate.

Although these examples of activity patterns in caudate neurons are observed for different tasks and during different task periods, they may all be thought of as reflecting a conversion of task-relevant information into a decision variable, to be then acted upon for the final selection. On the asymmetric reward task, relevant information includes external visual information, internally generated reward expectation for the visual target, and internally maintained knowledge about the current reward context. On the motion discrimination task, relevant information includes external visual evidence and idiosyncratic internal biases. The modulation patterns of caudate activity thus suggest a common selection process for different types of information. Interestingly, some dorsomedial striatal neurons in mice encode the net value of available actions, while another subpopulation encodes the relative value between alternative actions, the latter of which takes into account both olfactory sensory information and reward expectation (Wang et al., 2013). This idea receives further support from caudate activity dynamics: before target onset on the asymmetric reward task, the dynamics of caudate activity is consistent with accumulation of reward context information over time; during the early motion viewing period on the motion discrimination task, the dynamics of caudate activity is consistent with accumulation of motion evidence (Ding, 2015). In other words, caudate activity may reflect the adaptation of an accumulation-like function for different task-relevant inputs. Collectively, these results support the idea that the basal ganglia provide similar selection mechanisms for diverse task-relevant information.

## Striatal Activity Reflects Quantities Necessary for Evaluation

To achieve appropriate goal-directed behaviors, it is necessary to evaluate the selection process, online and/or after feedback, to guide adjustments if necessary. In the reinforcement learning framework, evaluation is implemented as a comparison between the predicted and received outcome. Consistent with the proposed roles of the basal ganglia in generating outcome predictions (Barto, 1995; Houk et al., 1995), neural activity reflecting the predicted and/or received outcomes is prevalent in the striatum.

**Figure 2:**
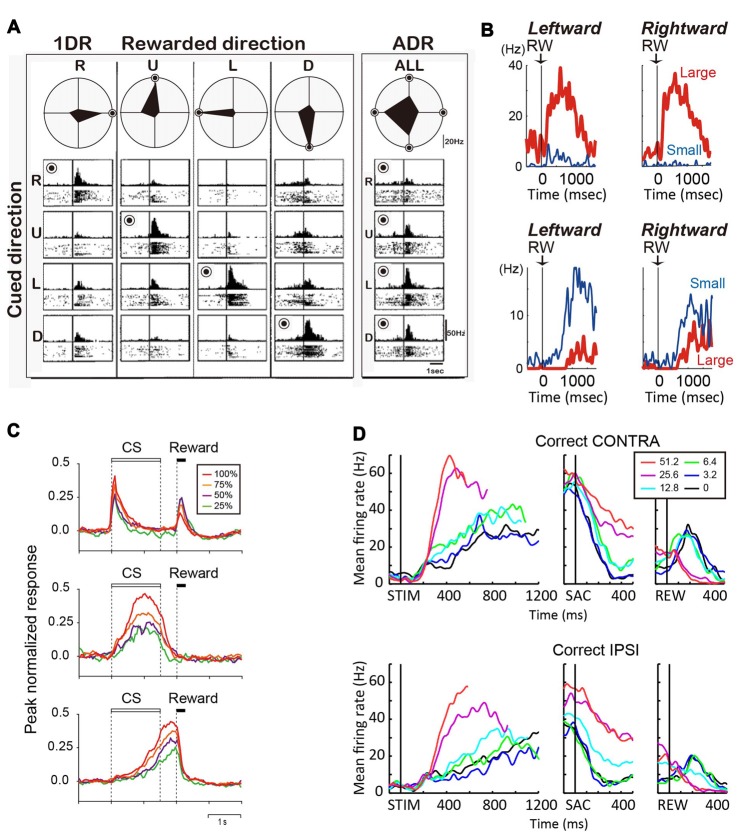
**(A)** Reward-dependent visual response of a right caudate neuron. The data obtained in one block of all-direction rewarded (ADR condition, right) and four blocks of one-direction rewarded (1DR condition, left) are shown in columns. The histograms and rasters are aligned on cue onset for different cue directions (R, right; U, up; L, left; D, down). The rewarded direction is indicated by a “bull’s eye mark”. Polar diagrams show the magnitudes of response for four cue directions. The neuron’s response was strongest for the rewarded direction in any block of 1DR, whereas its preferred direction was to the left in ADR (modified from Kawagoe et al., 1998). **(B)** Top, an example of central caudate neuronal activity showing a positive reward effect in the biased-reward saccade task (Figure 1A). Bottom, an example dorsal caudate neuron showing a “negative” reward effect. **(C)** Average activity of three types of neurons in the dorsal striatum of rats participating in a probabilistic Pavlovian conditioning task, in which auditory conditioned stimuli (CS) indicate reward probability. Top, CS phasic neurons; middle, CS tonic neurons; bottom, US build-up neurons. **(D)** An example neuron showing similar coherence modulation for both contra- and ipsi-lateral choices. Note the positive coherence modulation during dots viewing (STIM) and around saccade onset (SAC), and negative coherence modulation after reward onset (REW).

In tasks that explicitly manipulate reward outcomes, such as the asymmetric reward task described above, many caudate neurons encode the reward expectation, regardless of the target identity/location (Kawagoe et al., 1998; Takikawa et al., 2002). For example, the neuron in Figure 2A shows larger activation for targets paired with the larger reward. Such neurons often show remarkable task context dependance: when all targets were rewarded equally (“all-direction rewarded, ADR” in Figure 2A), the same neurons displayed clear selectivity for target location. In other words, in an equal-reward task context, these neurons may contribute to selection of the correct saccade target, by encoding target location information; however, in the asymmetric-reward task context (“1DR” in Figure 2A), the loss of target-location selectivity diminishes their usefulness for the selection process, while the emergence of reward expectation-selectivity increases their usefulness in monitoring a predicted reward, which is a critical component for evaluation of the selection process. Such signals tended to have a positive relationship with reward expectation, with the majority of the caudate neurons showing higher activity for larger reward expectation (Kawagoe et al., 1998). It should be noted, that although such signals occur at similar times as selection-related signals, the lack of choice specificity means that they cannot be used directly to select an action. Anatomically, these neurons are distributed in the dorsolateral part of caudate.

During the reward period (or after feedback), activity of many caudate neurons reflect the actual outcome (Hikosaka et al., 1989c; Lau and Glimcher, 2007, 2008; Nakamura et al., 2012). On the asymmetric reward task, the neuron in Figure 2B top panel was more active after a large reward, rather than a small reward. Conversely, the neuron in Figure 2B bottom panel was more active after a small than a large reward (Nakamura et al., 2012). Similar neurons are also reported for a probabilistic reward task (Lau and Glimcher, 2007). Neurons with small-reward preferring post-reward activity are widely distributed, while those with large-reward preferring post-reward activity are more concentrated in the rostral-ventral portions of the caudate (Nakamura et al., 2012).

Similar to the monkey caudate, the dorsomedial striatum in rodents also contains many neurons showing evaluative activity. For example, using a probabilistic reward Pavlovian conditioning task, Oyama et al. observed three types of modulation of striatal activity (Oyama et al., 2010, 2015). “Conditioned stimuli (CS) phasic neurons” showed higher phasic CS response and lower US response for larger reward probability (Figure 2C, top). “CS tonic neurons” showed higher tonic CS response for larger reward probability (Figure 2C, middle). “US buildup neurons” showed gradually increasing activity toward the time of reward delivery, with higher buildup response for higher reward probability (Figure 2C, bottom). In aggregate, these neurons exhibited responses to reward predicting cue (target or CS) that were positively correlated with reward prediction.

Reward prediction-related evaluative signals were also observed in monkey caudate nucleus in the motion discrimination task, in which reward expectation is not explicitly manipulated or signaled to the monkey but may be estimated based on the quality of the motion evidence. Evaluative activity on the motion discrimination task thus show features reminiscent of those observed on tasks with explicit reward manipulations (Ding and Gold, 2010): such activity is more strongly modulated by motion coherence than by motion direction or choice; activity observed after reward onset tends to be higher for low-coherence trials (i.e., trials with low reward expectation). For example, the neuron in Figure 2D shows higher activity for higher coherence trials for both contra- and ipsi-lateral motion directions/choices during the motion viewing and peri-saccade epochs. After reward, the sign of coherence modulation reversed for both choices, with higher activity observed for low-coherence trials. These neurons with evaluative signals were distributed in the same general caudate region as neurons with selection-related signals (Ding and Gold, 2010).

Thus, similar to selection-related activity, evaluation-related activity is also observed for different behavioral tasks. Such activity may be thought of as reflecting a conversion of task-relevant information into an estimate of reward expectation, to be then acted upon for evaluating how well the behavior achieves the subject’s goal. These results thus support the idea that the basal ganglia provide similar evaluation mechanisms for diverse task-relevant information.

## Discussion

The well-known heterogeneity in striatal activity can be described in multiple dimensions, such as cell type, macroscopic anatomical loops, nature of inputs, and context dependance. We have focused this brief review on putative projection neurons within a macroscopic loop (caudate in monkey/dorsomedial striatum in rodent) on two types of tasks, with explicit reward or visual input manipulations, respectively. With the obvious caveats associated with a limited survey of the vast literature of striatum, we hope to have illustrated a functional dimension to help “taming” the seemingly overwhelming striatal heterogeneity. Guided by previous insights on the modular organization and general selection-related functions of the basal ganglia, we have parsed the diverse striatal activity patterns into two functional categories: implementation of selection and evaluation.

Following this parsing scheme, we highlight several open questions. First, what is the relationship between this functional parsing and the anatomical partitioning of the caudate nucleus? The primate caudate is divided into “dorsal” and “ventral” subregions at the lower edge of the lateral ventricle, with the “ventral” caudate located at the furthest ventromedial location (Haber and Knutson, 2010; Cai et al., 2011). Neurons with selection-related activity, such as those modulated by block-wise reward context, target location, choice and/or motion evidence, are more prevalent in the dorsal than the ventral caudate in monkeys; neurons with evaluation-activity that prefer larger reward are more prevalent toward the ventral caudate; neurons with evaluation-related activity that prefer smaller reward are distributed more evenly. Similar functional distributions along the dorsal-ventral axis have also been observed in humans and rodents (O’Doherty et al., 2004; Atallah et al., 2007; Ito and Doya, 2009; Roesch et al., 2009; Cai et al., 2011). Such functional distributions seem to accord with extensive tracing results, which demonstrate a general sensory/motor-associative-limbic gradient along the dorsal-ventral axis in the cortical/subcortical inputs to caudate subregions, as well as the tripartite subdivisions defined by calbindin immunoreactivity in humans (Kelley et al., 1982; Selemon and Goldman-Rakic, 1985; Groenewegen et al., 1987; Heimer et al., 1987; Alexander and Crutcher, 1990a; Saint-Cyr et al., 1990; Yeterian and Pandya, 1991; Parent and Hazrati, 1994; Eblen and Graybiel, 1995; Haber and McFarland, 1999; Haber et al., 2000, 2006; Karachi et al., 2002; Haber, 2003; Haber and Knutson, 2010; Parker et al., 2016). It remains to be elucidated how these anatomical differences contribute to the response heterogeneity among striatal neurons. The striatum can also be divided into the direct and indirect pathways. To the extent that is tested, striatal neurons in both pathways show similar task modulation, suggesting that the parsing by direct-indirect pathways is orthogonal to the parsing by selection-evaluation (Cui et al., 2013; Barbera et al., 2016).

Second, is the selection vs. evaluation parsing maintained across tasks, at the single-neuron level? At the population level, there may be two non-overlapping pools of neurons responsible for selection and evaluation. Within a pool, different subsets of neurons in the two pools may participate in different tasks. It remains an open question whether the same neurons encode selection/evaluation-related signals on both asymmetric reward and perceptual decision tasks. Alternatively, given that neurons with evaluative activity on the asymmetric reward task can show robust spatial selectivity on an equal reward task, the selection-evaluation distinction may be considerably task-dependent (Kawagoe et al., 1998). In this case, neurons participating in selection for one task may participate in evaluation for another task.

Third, how stable is the selection vs. evaluation parsing during learning? Our survey focused on neural responses in well-trained animals. However, neural ensembles in the rat dorsomedial striatum and monkey dorsal striatum display striking changes during different stages of learning, particularly in cue presentation, choice and post-decision epochs (Thorn et al., 2010; Antzoulatos and Miller, 2011; Thorn and Graybiel, 2014). It is interesting to speculate whether single neurons switch between contributing to selection or to evaluation during training. If we assume that neural ensembles encoding selection and evaluation are stable, how do they interact with each other? Simultaneous recordings of multiple striatal neurons on reward-based or perceptual decision tasks could shed light on this question.

Lastly, although we focused this minireview article on the oculomotor and associative striatum, the basic parsing scheme may extend beyond that. For example, action value-related activity has also been observed in the monkey putamen (Samejima et al., 2005). The ventral striatum is generally thought to serve evaluative roles (“critic”), but is also known to exhibit heterogeneous signals that may be used for selection (e.g., van der Meer et al., 2010). In addition, selection- and/or evaluation-related striatal neurons could operate at different hierarchical levels such as the general goal (e.g., foraging vs. mating), sensory modality, effector and specific movement (e.g., left vs. right). If such neurons do contribute to a proposed “central selection” function of the basal ganglia, how are they coordinated across levels to achieve the final goal (Redgrave et al., 1999).

To summarize, parsing with computation categories, such as selection vs. evaluation, encourages a focus on the functional commonalities revealed by studies with different animal models and behavioral tasks, instead of a focus on aspects of striatal activity that may be specific to a particular task setting. Such a parsing may prove useful for exploration of striatal contributions to goal-directed behaviors.

## Author Contributions

LD and KN discussed and wrote the manuscript.

## Conflict of Interest Statement

The authors declare that the research was conducted in the absence of any commercial or financial relationships that could be construed as a potential conflict of interest.
